# A Machine Learning Model Based on Clinical Factors to Predict the Efficacy of First-Line Immunochemotherapy for Patients With Advanced Gastric Cancer: Retrospective Study

**DOI:** 10.2196/82533

**Published:** 2025-12-22

**Authors:** Xu Cheng, Ping Li, Enqing Meng, Xinyi Wu, Hao Wu

**Affiliations:** 1 Gastric Cancer Center The First Affiliated Hospital of Nanjing Medical University Nanjing China; 2 Department of Oncology The First Affiliated Hospital of Nanjing Medical University Nanjing China

**Keywords:** advanced gastric cancer, immunochemotherapy, machine learning, random survival forest, prognostic models

## Abstract

**Background:**

The development of immunotherapy has provided new hope for patients with advanced gastric cancer (AGC). However, due to the high heterogeneity of the disease, the efficacy of first-line immunochemotherapy varies among patients. There is still a lack of simple and effective models to predict the efficacy of immunochemotherapy in this setting.

**Objective:**

This study aimed to identify critical factors and develop predictive models to evaluate the efficacy of first-line immunochemotherapy in patients with AGC using clinically available data. The goal was to offer evidence-based guidance for clinical practice and enable personalized treatment strategies.

**Methods:**

To evaluate the effectiveness of first-line immunochemotherapy in AGC, we retrospectively collected clinical data from The First Affiliated Hospital of Nanjing Medical University between January 2018 and October 2023. The data collected were divided into a training set (168/240, 70%) and an internal validation set (72/240, 30%). Additionally, a temporal validation cohort of 76 patients recruited from November 2023 to September 2024 was assembled to further evaluate the predictive performance of the models. We used univariate and multivariate Cox regression analyses, along with the least absolute shrinkage and selection operator (LASSO) regression, and integrated clinical expertise to identify key predictors of treatment efficacy and to construct the LASSO-Cox model. We developed 4 models (LASSO-Cox, random survival forest [RSF], extreme gradient boosting, and survival support vector machine) and evaluated their performance using the C-index, area under the curve (AUC), calibration curves, and decision curve analysis. The optimal model was interpreted using Shapley additive explanations, and its risk scores were used to stratify patients for Kaplan-Meier survival analysis.

**Results:**

Among the 4 prognostic models developed in this study, the RSF model demonstrated superior predictive accuracy and discrimination for progression-free survival, as evidenced by its higher AUC, concordance index, continuous AUC curves, and calibration curves compared with the other 3 models. Additionally, decision curve analysis showed that the RSF model offered greater net clinical benefit. The Shapley additive explanations results identified that age, histological subtype, the proportion of CD19^+^ B cells, CD16^+^CD56^+^ natural killer cells, and the presence of liver metastasis were key prognostic factors influencing patient outcomes. Patients in the low-risk group, as determined by the RSF model’s risk score, exhibited a significantly higher progression-free survival rate than those in the high-risk group, further validating the value of the RSF model for risk stratification.

**Conclusions:**

This study is the first to use machine learning algorithms to develop a predictive model for the efficacy of first-line immunochemotherapy in AGC, and to identify key predictors of treatment outcome. The results indicate that the RSF model not only enables precise stratification of patients likely to benefit but, more importantly, provides quantifiable decision support for individualized clinical strategies, underscoring its potential value in clinical decision-making.

## Introduction

Gastric cancer (GC) ranks as the fifth most common malignancy worldwide and is the fourth leading cause of cancer-related mortality [1]. In 2022, there were over 968,000 new cases of GC, resulting in nearly 660,000 deaths [2]. Due to the delayed onset of clinical symptoms and the absence of effective diagnostic methods for early-stage GC, 40% of patients are diagnosed at an advanced stage, where surgical intervention is typically not feasible [3]. Among patients undergoing radical resection, over two-thirds experience recurrence or metastasis [4,5]. Consequently, chemotherapy remains the cornerstone of treatment for advanced gastric cancer (AGC), yet the prognosis for advanced or metastatic cases remains dismal, with an overall survival (OS) of only 9-14 months [6]. Therefore, a comprehensive upfront assessment of prognosis is essential for guiding clinical decisions in multidisciplinary treatment approaches [7].

The advent of immune checkpoint inhibitors (ICIs), particularly those targeting programmed cell death protein 1 (PD-1) and programmed death-ligand 1 (PD-L1), has revolutionized cancer therapy, yielding robust and durable responses in GC. Clinical trials, such as CheckMate-649 and KEYNOTE-062, have demonstrated that combining immunochemotherapy significantly improves OS and progression-free survival (PFS) in patients with PD-L1–positive tumors [8,9]. However, the high heterogeneity of GC leads to variations in the efficacy of immunochemotherapy among different patients [10]. Identifying individuals who are likely to respond to immunochemotherapy is therefore critical for providing personalized treatment.

Nowadays, numerous biomarkers, including tumor mutation burden (TMB), PD-L1 expression, microsatellite instability (MSI), and Epstein-Barr virus infection status, have been proposed to predict responsiveness to PD-1 and PD-L1 inhibitors [11,12]. Additionally, circulating tumor DNA positivity has been associated with relapse and poorer prognosis [13]. However, given the highly heterogeneous nature of GC, relying on a single factor for prognostic prediction is often insufficient [14]. For instance, only 45% of patients with high TMB (≥20 mutations per megabase [mut/Mb]) respond to immunotherapy, while approximately 5% of patients with low TMB exhibit significant therapeutic responses to ICIs [15,16]. Furthermore, the predictive utility of single biomarkers is undermined by the dynamic alteration of the tumor microenvironment following therapeutic interventions (eg, chemotherapy or radiotherapy), as well as inconsistencies in detection methodologies and threshold definitions—such as the varying PD-L1 scoring systems (tumor proportion score, combined positive score, immune cell score, and H-score) and platform-dependent criteria for MSI and TMB. While recent studies have developed prognostic models based on transcriptomics or targeted metabolomics [17-19], the clinical translation of these omics-based signatures is severely constrained by their high costs, complex data processing, and reliance on specialized laboratory infrastructure [20,21]. This creates a clear and unmet need for prognostic tools built on more accessible, cost-effective, and routinely collected data. Although recent studies have made progress in applying machine learning (ML) algorithms to clinical data for postoperative patients with GC or to predict immunotherapy-related adverse events in AGC [22,23], a critical gap remains, as robust predictive models for the efficacy of first-line immunochemotherapy in the advanced setting are notably lacking. Therefore, this study was designed to address this specific gap. We aimed to develop and validate a robust prognostic model based only on readily available baseline clinical parameters. The objective of this tool is to effectively predict first-line immunochemotherapy outcomes, identify patients most likely to benefit, and thereby advance personalized therapy for AGC. Furthermore, understanding the key risk factors derived from such a model is crucial for devising appropriate follow-up strategies.

The Cox proportional hazards model has long been the standard for quantifying the impact of clinical indicators on survival outcomes [24,25]. However, its reliance on linear assumptions often fails to capture the intricate, nonlinear patterns characteristic of heterogeneous diseases like GC. Consequently, there is a need for more adaptive modeling strategies to maximize predictive performance. To this end, we used a comprehensive ML framework comprising random survival forest (RSF), extreme gradient boosting (XGBoost), and survival support vector machine (SVM). These algorithms were selected for their specific strengths in handling complex survival data: RSF is robust in ranking variable importance [26,27]; XGBoost offers superior efficiency and the ability to model complex feature interactions; and survival-SVM excels in distinguishing risk stratifications in high-dimensional spaces [28,29]. Our primary objective was not merely to compare these methodologies, but to leverage their combined strengths to select the best-performing model. This approach ensures the development of a clinically practical and highly accurate prognostic instrument for first-line immunochemotherapy [30-32].

Given the poor prognosis and high recurrence rate of AGC, as well as the urgent need for individualized treatment strategies, the ability to predict risk factors associated with prognosis and survival rates before treatment could enable more targeted therapeutic adjustments, ultimately improving patient outcomes and quality of life. This study aims to retrospectively analyze the clinical and pathological data of patients with AGC who received first-line immunochemotherapy. By identifying key prognostic factors that predict the efficacy of immunochemotherapy, 4 prognostic models will be developed and compared in terms of discrimination, calibration, stability, and clinical net benefit. The optimal model will be selected for clinical application, providing a reference for personalized treatment strategies and ultimately improving the prognosis of patients with AGC.

## Methods

### Data Source and Study Population

Clinical data of patients for the model development cohort were collected retrospectively from January 1, 2018, to October 31, 2023, at The First Affiliated Hospital of Nanjing Medical University, China. Additionally, a temporal validation cohort between November 1, 2023, and September 13, 2024, was included from the same hospital. Patients were enrolled in this study based on the following inclusion criteria: (1) aged 18 years or older, (2) histologically or cytologically confirmed gastric adenocarcinoma, (3) received ICIs as first-line treatment for advanced disease, (4) presence of at least 1 measurable lesion according to the Response Evaluation Criteria in Solid Tumors (RECIST) version 1.1. (lesions in hollow viscera, such as the esophagus and stomach, were not considered measurable; lesions situated in a previously irradiated area were considered measurable only if progression had been demonstrated), and (5) Eastern Cooperative Oncology Group performance status of 0 or 1. The exclusion criteria were (1) discontinuation of treatment due to intolerance to immunotherapy or combination agents, (2) receiving fewer than 2 treatment cycles, precluding efficacy assessment, (3) presence of concurrent malignancies of other origins, and (4) incomplete baseline clinical data or lost to follow-up.

### Data Collection and Outcomes

Baseline characteristics were assessed, including age, sex, BMI, presence of underlying comorbidities, Eastern Cooperative Oncology Group performance status, smoking history, drinking history, tumor location (including cardia and esophagogastric junction, fundus, body, antrum, and pylorus of the stomach), Lauren classification (including intestinal type, mixed type, diffuse type, and unknown), tumor differentiation (including G3, G2, G2-G3, and unknown), histopathological type (including gastric adenocarcinoma and signet ring cell carcinoma), intraperitoneal chemotherapy (with or without), type of PD-1 or PD-L1 inhibitor (including sintilimab, nivolumab, tislelizumab, camrelizumab, and others), radiotherapy (with or without), targeted therapy (with or without), use of antiangiogenic drugs (including anlotinib, apatinib, others, and none), pretreatment levels of alpha-fetoprotein (AFP; <1.9 or ≥1.9 ng/μL), the pretreatment percentage of CD16^+^CD56^+^ natural killer (NK) cell (<27.41 or ≥27.41), the pretreatment percentage of the ratio of CD4^+^ to CD8^+^ T cell (<2.66 or ≥2.66), the pretreatment percentage of CD19^+^ B cell (<12.04 or ≥12.04), human epidermal growth factor receptor 2 (HER2) expression status (including positive, negative, and unknown), PD-L1 expression status (including positive, negative, and unknown), microsatellite stability (including microsatellite stability, high MSI, and unknown), TMB status (including low, high, and unknown), presence of liver metastasis (with or without), peritoneal metastasis (with or without), bone metastasis (with or without), and distant organ metastasis (with or without).

To ensure data consistency and reliability, all data collection adhered to strict standards. Tumor location, differentiation grade, Lauren classification, and histopathological type were categorized based on the 2024 guidelines of the Chinese Society of Clinical Oncology. BMI was classified according to the World Health Organization criteria, with a BMI ≥25 considered overweight. Continuous numerical variables, including age, pretreatment levels of AFP, and pretreatment lymphocyte subset percentages, were subsequently categorized into discrete groups by determining optimal cutoff values in order to enhance analytical convenience and effectiveness. HER2 expression status was determined through immunohistochemistry or fluorescence in situ hybridization: 0 to 1+ was classified as negative; 2+ was considered equivocal, requiring confirmation via fluorescence in situ hybridization; and 3+ was classified as positive. PD-L1 expression status was assessed following the standards of the US Food and Drug Administration: PD-L1 combined positive score <1 was classified as negative; PD-L1 combined positive score ≥1 was classified as positive. Microsatellite stability status was defined as follows: microsatellite stability indicated no significant difference in microsatellite length between tumor and normal tissue; high MSI was defined as the presence of insertion or deletion mutations in at least 2 microsatellite loci. TMB≥10 mut/Mb was classified as high expression; TMB<10 mut/Mb was categorized as low expression. AFP and lymphocyte subset data were obtained from peripheral blood samples collected before first-line immunochemotherapy in patients with AGC. Distant metastasis and disease progression were evaluated through contrast-enhanced computed tomography scans of the chest and entire abdomen. Progressive disease was determined based on the RECIST version 1.1. The primary end points of this study were the predictive capability, validity, and clinical utility of the developed prognostic model. The primary time-to-event outcome was PFS, defined as the interval from the initiation of first-line immunochemotherapy to the date of radiologically confirmed disease progression. The model development cohort was followed up until March 13, 2024, while the temporal validation cohort was followed up until September 13, 2024. Progressive disease was assessed via imaging evaluations conducted every 2 treatment cycles, ensuring that all outcome assessments were supported by radiographic evidence. This methodology ensures the robustness and clinical applicability of the prognostic models developed in this study.

### Statistical Analysis

The dataset for the model development cohort was split into a training cohort (168/240, 70%) and a validation cohort (72/240, 30%). The training cohort was used for model construction, while the training cohort and the internal validation cohort were used for model assessment. Additionally, a temporal validation cohort was used to further assess model performance over time as an external validation set. Categorical variables are presented as frequencies and percentages, with group comparisons made using the chi-square test or Fisher exact test. Normally distributed continuous variables are expressed as the mean and SD, and between-group comparisons are performed using the 2-tailed *t*-test. Nonnormally distributed continuous variables are reported as the median and IQR, with between-group comparisons conducted using the Wilcoxon rank-sum test. Statistical significance is defined as *P*<.05.

All baseline clinical characteristics were incorporated into the construction of the prognostic model. Patients with missing data rates ≥10% for baseline characteristics were excluded from the analysis. For cases with missing values <10%, we used multiple imputation by chained equations, implemented with the “mice” R package. The multiple imputation by chained equations procedure was configured to generate five imputed datasets (m=5), with a fixed random seed to ensure reproducibility. The imputation model included predictor variables (the pretreatment proportions of CD16^+^CD56^+^ NK cells, CD19^+^ B cells, and CD4^+^/CD8^+^ T cells) and the outcome variable (PFS and event status) to preserve prognostic correlations. For continuous variables, the default predictive mean matching method was used, which is robust to nonnormality. This advanced approach iteratively generates multiple imputed datasets through observed data patterns while appropriately accounting for the uncertainty inherent in the imputation process. After imputation, we generate diagnostic plots to examine the distribution of the imputed data (Multimedia Appendix 1). The results indicated that, for most variables, the imputed values aligned well with the original data distribution. Because missing data patterns may influence study outcomes, we conducted 2-tailed *t*-tests and Kolmogorov-Smirnov (KS) tests on both the imputed and original complete datasets to assess any significant differences. The pretreatment proportion of CD16^+^CD56^+^ NK cells had 2-tailed *t*-test and KS test *P* values of .73 and .98, respectively, before and after imputation; the pretreatment proportion of CD19^+^ B cells showed 2-tailed *t*-test and KS test *P* values of .51 and .99, respectively; and the pretreatment proportion of CD4^+^/CD8^+^ T cells had 2-tailed *t*-test and KS test *P* values of .60 and .99, respectively. None of these variables demonstrated a statistically significant difference (*P*>.05), suggesting that the chosen imputation method had minimal impact on the overall analysis. Furthermore, to assess the robustness of our results and rule out potential bias introduced by the imputation of missing values, a sensitivity analysis was performed. We reconstructed and re-evaluated all 4 models using a complete-case cohort (excluding all patients with missing data). The results from this analysis were consistent with those derived from the imputed dataset, indicating that the imputation procedure introduced negligible bias to the model performance (Multimedia Appendix 2).

In this study, important predictive features were identified through a combination of univariate analysis, least absolute shrinkage and selection operator (LASSO) regression, multivariate analysis, and the clinical expertise of 2 experienced clinicians, which served as the foundation for constructing a LASSO-Cox model. In parallel, ML was used to develop RSF, XGBoost, and survival-SVM models, with parameter tuning carried out for each. The performance of these 4 models was systematically evaluated and compared using metrics such as area under the curve (AUC), concordance index (C-index), receiver operating characteristic (ROC) curves, continuous AUC curves, calibration curves, and decision curve analysis (DCA) to identify the optimal predictive model. Patients were then stratified into high- and low-risk groups based on the risk scores derived from this optimal model, and Kaplan-Meier (KM) analysis was performed to assess survival differences. Finally, Shapley additive explanations (SHAP) analysis was conducted to interpret the optimal model and to pinpoint the key predictive variables. All statistical analyses were performed using R version 4.4.1 (R Foundation for Statistical Computing).

### Ethical Considerations

This study was approved by the Ethics Committee of The First Affiliated Hospital of Nanjing Medical University (approval 2024-SR-453). No personally identifiable information was involved in this study. Due to the retrospective nature of the research, the requirement for informed consent was waived.

## Results

### The Clinical Characteristics of Patients With AGC

**Figure 1:**
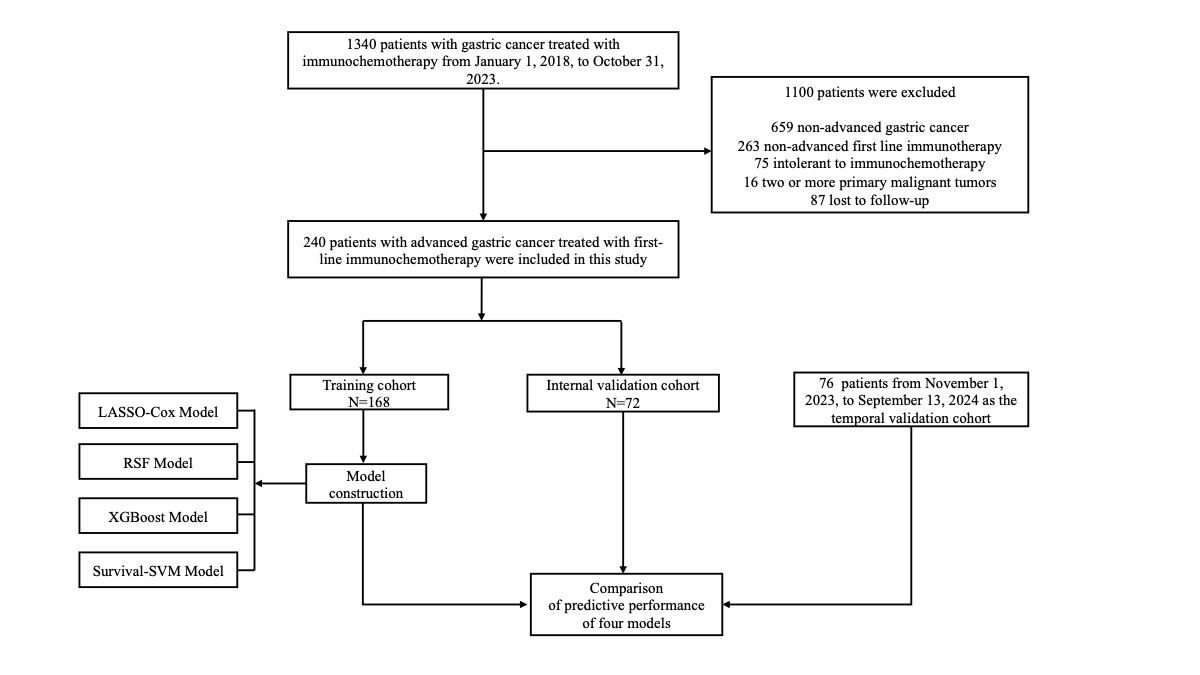
Study flow chart. LASSO: least absolute shrinkage and selection operator; SVM: support vector machine; RSF: random survival forest; XGBoost: extreme gradient boosting.

The workflow of the study is illustrated in Figure 1. Ultimately, 316 eligible patients were included in the study. Among them, 240 patients were enrolled between January 1, 2018 and October 31, 2023, forming the model development cohort, while the remaining 76 patients, recruited between November 1, 2023 and September 13, 2024, comprised the temporal validation cohort for further assessment of the model’s predictive performance. The median follow-up duration for the model development cohort was 16 (95% CI 13.1-18.9) months, with a median progression-free survival (mPFS) of 8.97 (95% CI 7.9-11.1) months. In the temporal validation cohort, the median follow-up time was 10.3 (95% CI 9.5-upper limit not reached) months. Patients in the model development cohort were randomly divided into a training set (n=168, 70%) and an internal validation set (n=72, 30%). Baseline clinical characteristics of all patients are summarized in Multimedia Appendix 3. The mPFS was 8.83 (95% CI 7.43-11.4) months in the training cohort and 10 (95% CI 7.77-15.6) months in the internal validation cohort. In the temporal validation cohort, the mPFS was 6.8 (95% CI 6.4-8.03) months. The 6-month, 12-month, and 18-month PFS rates in the training set were 61%, 25%, and 11%, respectively, while in the internal validation set, they were 57%, 26%, and 13%, respectively. In the temporal validation cohort, the 6-month, 9-month, and 12-month PFS rates were 53%, 17%, and 8%, respectively. Due to the limited follow-up period in the temporal validation cohort, the 18-month PFS rate was not determined.

### Construction of the LASSO-Cox Model and Nomogram

**Figure 2:**
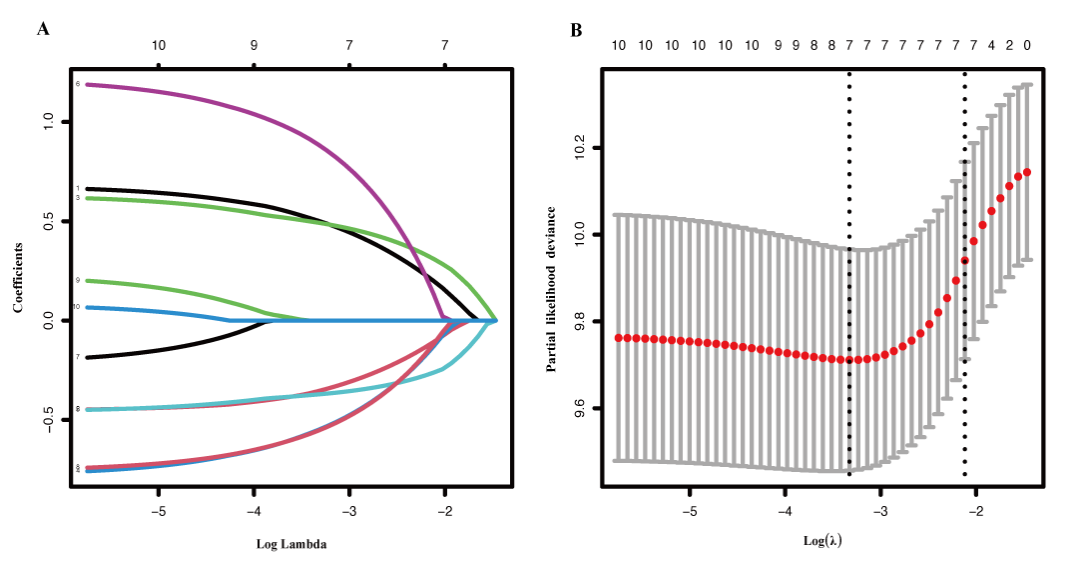
Feature selection using the least absolute shrinkage and selection operator regression. (A) The variation characteristics of the coefficient of variables; and (B) the selection process of the optimum value of the parameter λ in the least absolute shrinkage and selection operator regression model by cross-validation method.

The prognostic value of various factors for predicting PFS was assessed using the training cohort. In univariate analysis, age, histological subtype, pretreatment levels of AFP, pretreatment proportion of CD19^+^ B cell, CD16^+^CD56^+^ NK cell, CD4^+^/CD8^+^ T cell, TMB expression, and the presence of liver metastases were all found to be statistically significant (*P*<.05) (Multimedia Appendix 4). Recognizing that reliance solely on univariate analysis might introduce bias, 2 experienced clinicians reviewed these results and manually incorporated additional clinically relevant variables that, although not significant in the univariate analysis, have been linked to treatment efficacy, for example, PD-L1 expression, HER2 status, and MSI. This approach ensured that the model encompassed variables that are both clinically relevant and biologically meaningful. Subsequently, the significant factors from the univariate analysis were combined with clinically pertinent variables to form a LASSO regression. The LASSO algorithm was applied to identify the variables most strongly associated with PFS (Figure 2A). Through cross-validation, the optimal regularization parameter (λ) was determined to be 0.043, corresponding to the minimum partial likelihood deviance. Ultimately, 7 variables with nonzero coefficients were retained, that is, age, histological subtype, pretreatment levels of AFP, pretreatment proportion of CD19^+^ B cell, CD16^+^CD56^+^ NK cell, CD4^+^/CD8^+^ T cell, and the presence of liver metastases (Figure 2B).

**Figure 3:**
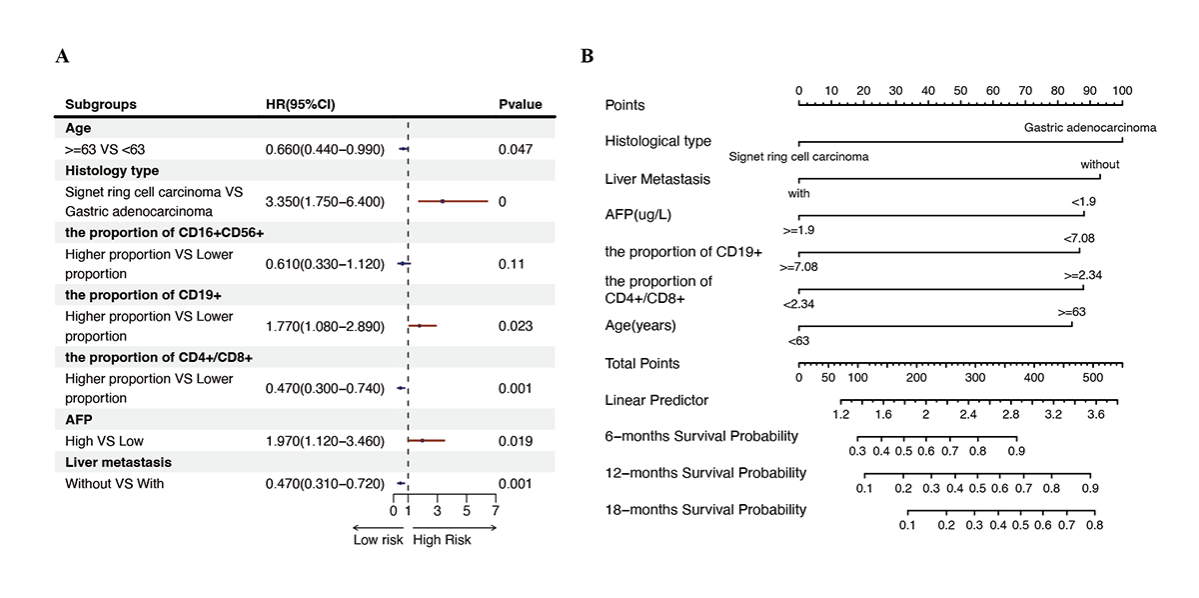
(A) Forest plot showing hazard ratios for different factors. (B) Nomogram for predicting progression-free survival of patients with advanced gastric cancer receiving first-line treatment with immunochemotherapy. AFP: alpha-fetoprotein.

To further evaluate the independent impact of the selected variables on the prognosis of AGC, a multivariate Cox regression analysis was performed based on the 7 variables identified through LASSO regression. Among these, age (*P*=.047), histological subtype (*P*<.001), pretreatment levels of AFP (*P*=.02), pretreatment proportion of CD19^+^ B cells (*P*=.02), CD4^+^/CD8^+^ T cells (*P*=.001), and liver metastasis (*P*=.001) were identified as independent predictors of PFS in patients with AGC (Table 1). The forest plot of the multivariate Cox regression analysis is illustrated in Figure 3A. Subsequently, the 6 independent predictors determined by the multivariate analysis were incorporated into a nomogram model for visual representation. In the nomogram, each variable is assigned a specific weight, and scores for individual predictors are projected onto a total points axis. The cumulative score is then used to estimate the 6-, 12-, and 18-month PFS rates by drawing a vertical line from the total points to the outcome axis (Figure 3B).

**Table 1:** Results of multivariate Cox proportional hazards regression analysis for progression-free survival in the training set.

Characteristics		Multivariable Cox regression, hazard ratio (95% CI)	*P* value
**Age (years)**			
	≥63 vs <63	0.660 (0.44-0.99)	.047
**Histological type**			
	Signet ring cell carcinoma vs gastric adenocarcinoma	3.35 (1.75-6.40)	<0.001
**AFP (ng/μL)**			
	≥1.9 vs <1.9	1.97 (1.12-3.46)	.02
**The proportion of CD16^+^CD56^+^T cell**			
	≥27.41 vs <27.41	0.61 (0.33-1.12)	.110
**The proportion of CD19^+^B cell**			
	≥12.04 vs <12.04	1.77 (1.08-2.89)	.02
**The proportion of CD4^+^/CD8^+^T cell**			
	≥2.66 vs <2.66	0.47 (0.30-0.74)	.001
**Liver metastasis**			
	Without vs with	0.47 (0.31-0.72)	.001

### Construction of Prognostic Models Based on ML

To establish a robust predictive model, PFS was categorized into short-PFS and long-PFS groups based on the cut point value determined in the training cohort. A decision tree (DT) model was first developed using the training cohort and validated on the internal validation cohort. However, the DT model demonstrated suboptimal performance (accuracy=0.5278). Given the limitations of the DT model described above, we turned to a more robust ML method—the RSF model. In the training cohort, we used 5-fold cross-validation and grid search to optimize the model’s hyperparameters. The grid search spanned a range of values for “mtry” (1 to 30) and “nodesize” (1 to 50), and the number of trees (“ntree”) was ultimately fixed at 1000. When “mtry” (the number of variables considered at each split) was set to 9 and “nodesize” (the minimum sample size in terminal nodes) was set to 6, the model achieved an out-of-bag error rate of 37%. At this configuration, the model reached its maximum AUC, while the error rate remained low and stable (Multimedia Appendix 5). The RSF model presents the relationship between the trees and error rate, and the variable importance rankings and grid search process. The top 9 variables significantly influencing the model were age, pretreatment proportion of CD19^+^ B cells, liver metastasis, pretreatment proportion of CD16^+^CD56^+^ NK cells, histological subtype, pretreatment proportion of CD4^+^/CD8^+^ T cells, pretreatment levels of AFP, sex, and PD-L1 expression (Multimedia Appendix 6).

In addition, we developed XGBoost and survival-SVM models based on ML methods to comprehensively evaluate the performance of different algorithms for predicting prognosis in patients with AGC. For the XGBoost model, grid search and cross-validation were used to determine the optimal hyperparameters, and the model was constructed and fine-tuned on the training cohort. In contrast, the survival-SVM model leveraged the strengths of SVMs in handling survival data by optimizing kernel parameters to capture nonlinear relationships among samples. Subsequently, the predictive performance of these 4 models was compared to assess their robustness and practical applicability.

### Comparison of Predictive Performance of 4 Models

To evaluate the predictive performance of the models, we assessed their discrimination, calibration, and robustness at different time points (Table 2). The RSF and XGBoost models exhibit higher C-index values, suggesting stronger predictive capabilities. In both the internal validation cohort and the temporal validation cohort, the RSF model demonstrated significantly superior AUC values compared with the other 3 models, indicating its superior discriminatory power.

**Table 2:** The concordance index and area under the curve values for the 4 models in both the internal validation cohort and the temporal validation cohort.

Model	C-index^a^	AUC^b^					
		Internal validation cohort (n=72)			Temporal validation cohort (n=76)		
		6 months	12 months	18 months	6 months	9 months	12 months
RSF^c^	0.828	0.887	0.943	0.945	0.896	0.930	0.950
LASSO^d^-Cox	0.719	0.660	0.756	0.778	0.724	0.720	0.728
XGBoost^e^	0.836	0.743	0.634	0.605	0.771	0.792	0.663
Survival-SVM^f^	0.740	0.789	0.808	0.711	0.802	0.825	0.697

^a^C-index: concordance index.

^b^AUC: area under the curve.

^c^RSF: random survival forest.

^d^LASSO: least absolute shrinkage and selection operator.

^e^XGBoost: extreme gradient boosting.

^f^SVM: support vector machine.

**Figure 4:**
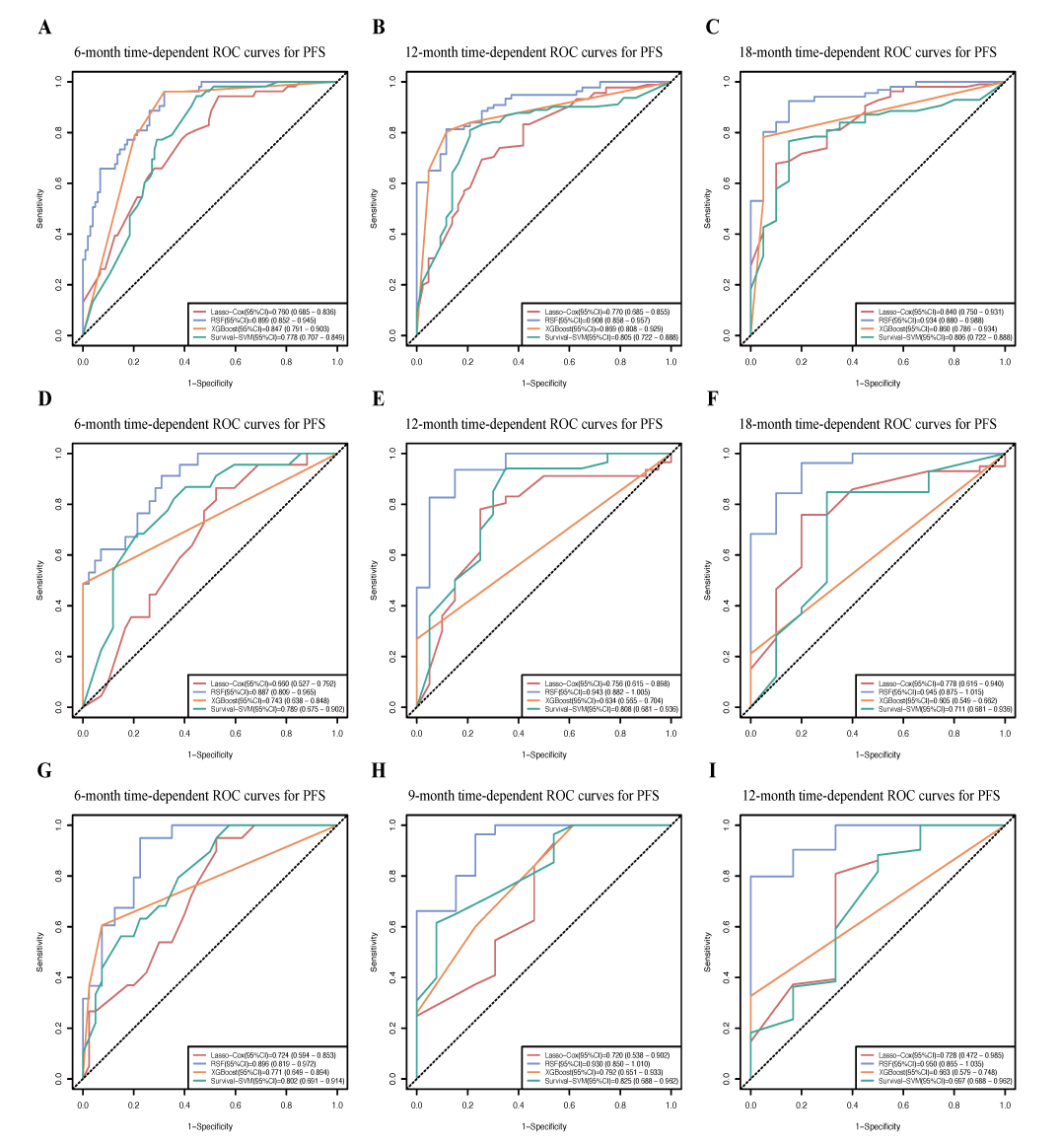
Comparison of time-dependent receiver operating characteristic curves of 4 models for predicting progression-free survival. (A-C) Receiver operating characteristic curves in the training cohort; (D-F) receiver operating characteristic curve in the internal validation cohort; and (G-I) receiver operating characteristic curves in the temporal validation cohort. Lasso: least absolute shrinkage and selection operator; PFS: progression-free survival; RFS: random survival forest; ROC: receiver operating characteristic; SVM: support vector machine; XGBoost: extreme gradient boosting.

Figures 4A-4F illustrate the ROC curves for predicting 6-, 12-, and 18-month PFS rates using 4 models in both the training and internal validation cohorts, while Figures 4G-4I display the ROC curves for predicting 6-, 9-, and 12-month PFS rates in the temporal validation cohort. To compare the performance of these models, we analyzed the differences in AUC values using the DeLong test. The results confirmed that the RSF model significantly outperformed the other 3 models at all time points (*P*<.05). Notably, the RSF model exhibited higher AUC values for 12- and 18-month PFS predictions across all cohorts, suggesting its strong potential in long-term survival prediction.

**Figure 5:**
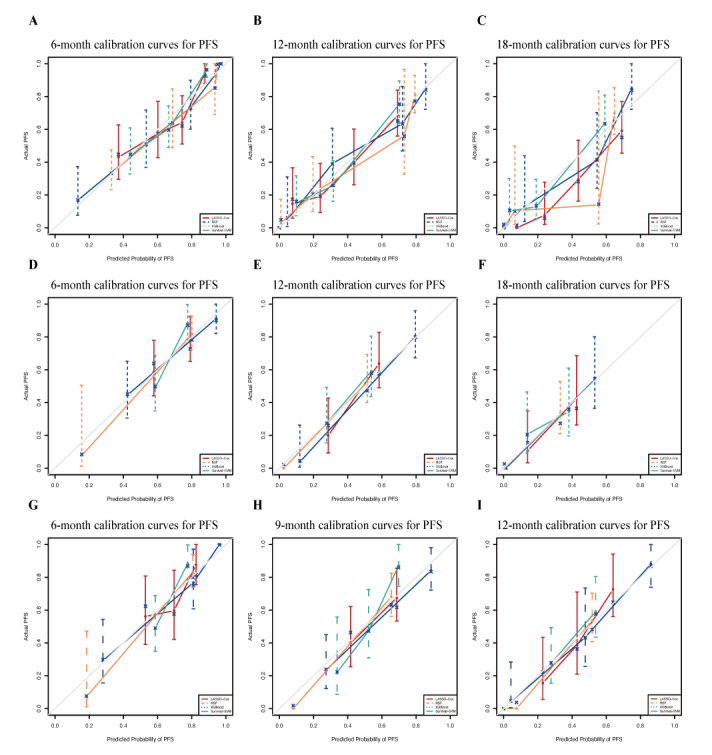
Calibration curves for the 4 models. (A-C) Calibration curves for the training cohort; (D-F) calibration curves for the internal validation cohort; and (G-I) calibration curves for the temporal validation cohort. LASSO: least absolute shrinkage and selection operator; PFS: progression-free survival; RFS: random survival forest; SVM: support vector machine; XGBoost: extreme gradient boosting.

To assess the accuracy of the predictive models for PFS rates, we generated calibration curves. The x-axis represents the predicted PFS values, while the y-axis shows the actual PFS. The calibration curves for the training, internal validation, and temporal validation cohorts were all close to the ideal line, demonstrating that all 4 models are well-calibrated and that there is strong concordance between the predicted and observed outcomes. This further supports the predictive accuracy of the RSF model (Figures 5A-5I).

Furthermore, the continuous AUC curves for the RSF model at different time points were consistently higher, further confirming its excellent and long-term discriminatory power in both the internal validation and temporal validation cohorts (Multimedia Appendix 7).

**Figure 6:**
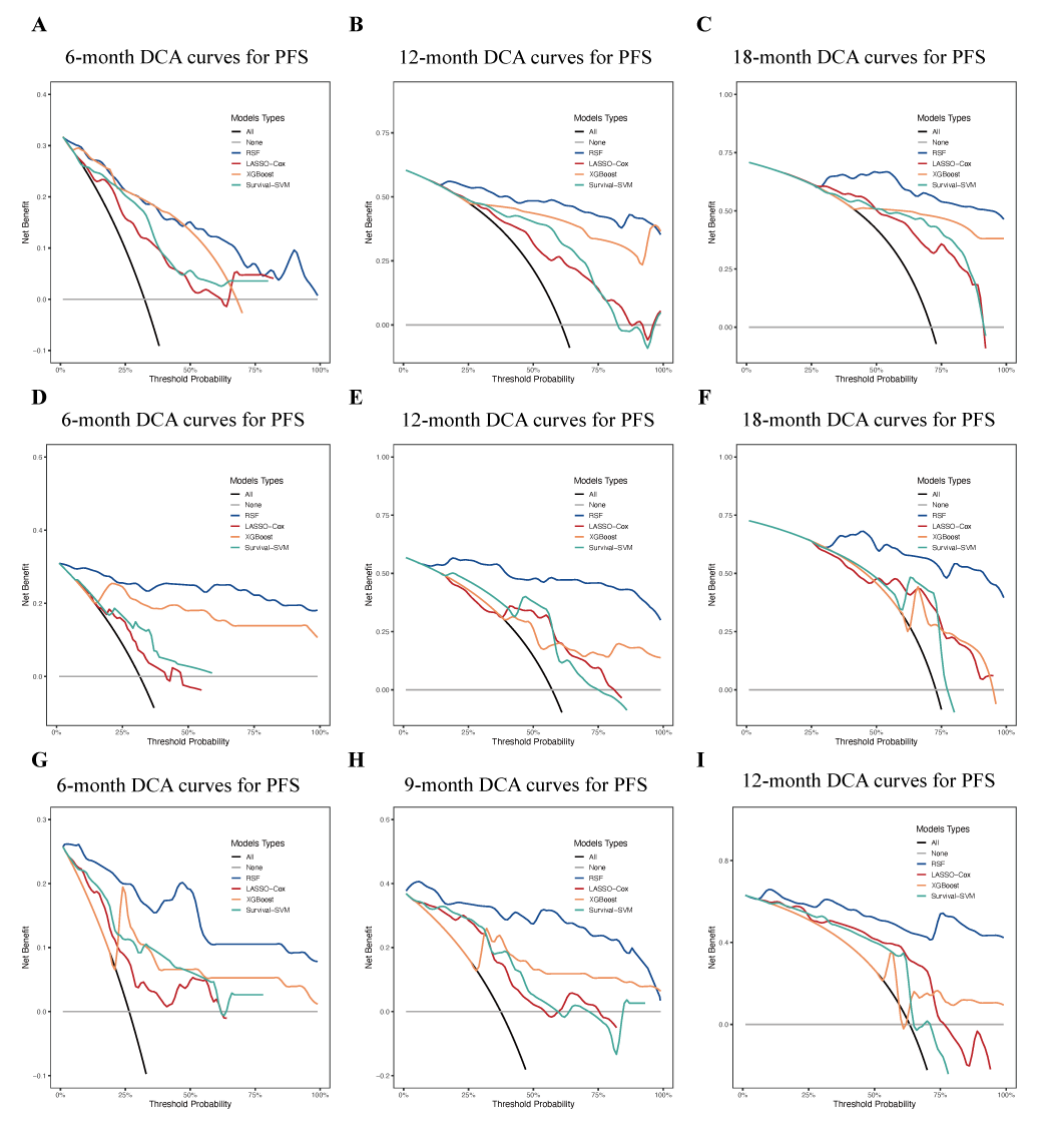
Decision curve analyses for the 4 models. (A-C) Calibration curves for the training cohort; (D-F) calibration curves for the internal validation cohort; and (G-I) calibration curves for the temporal validation cohort. DCA: decision curve analysis; LASSO: least absolute shrinkage and selection operator; PFS: progression-free survival; RFS: random survival forest; SVM: support vector machine; XGBoost: extreme gradient boosting.

DCA, which evaluates the clinical utility of models by calculating net benefits across various threshold probabilities, demonstrated that higher net benefits correspond to greater clinical decision-making value. The DCA curves of the 4 models revealed that the RSF model exhibited consistently superior net benefits compared with the other 3 models in all datasets, including the training set, internal validation set, and temporal validation cohort (Figure 6A-I).

**Figure 7:**
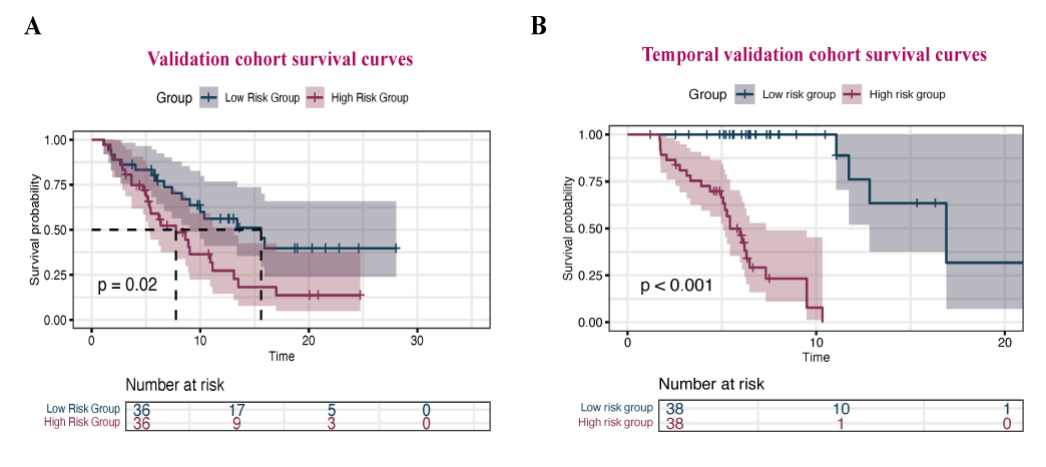
Kaplan-Meier survival curves of the high and low risk groups in internal validation cohort (*P*=.02) and temporal validation cohort (*P*<.001).

Based on comprehensive comparisons of the 4 models in discrimination, calibration, consistency, and clinical net benefit, our findings indicate that the RSF model demonstrates superior predictive performance compared with the other 3 models. Patient risk stratification constitutes a critical component in guiding clinical management decisions. Using risk scores calculated by the RSF model, the optimal cutoff value for stratifying the cohort into high-risk and low-risk groups was determined as 55.42. Subsequent stratification revealed distinct survival outcomes, as illustrated by risk score distribution plots and KM survival curves. The KM analysis demonstrated significantly higher survival rates in the low-risk group compared with the high-risk group across both internal validation and temporal validation cohorts (Figure 7). These findings were statistically consistent regardless of the evaluation time frame.

### Feature Importance Analysis

**Figure 8:**
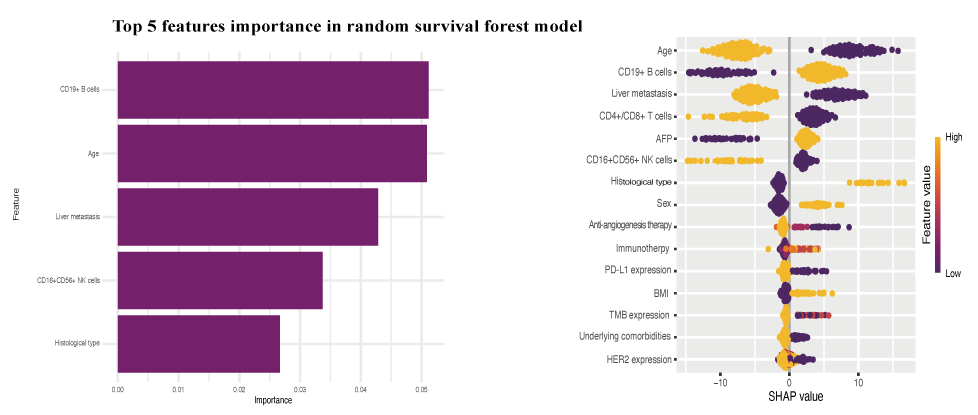
Feature importance ranking bar plot and Shapley Additive Explanations beeswarm plot of the random survival forest model. AFP: alpha-fetoprotein; HER2: human epidermal growth factor receptor 2; NK: natural killer; PD-L1: programmed death-ligand 1; TMB: tumor mutation burden.

To elucidate the decision-making rationale of the RSF model in predicting PFS for patients with AGC receiving first-line immunochemotherapy, interpretability analysis was performed using SHAP. Figure 8 displays the feature importance bar plot and SHAP value bee swarm plot for the RSF model. The analysis revealed that features including age, histological subtype, pretreatment proportion of CD19⁺ B cells, pretreatment proportion of CD16⁺CD56⁺ NK cells, and presence of liver metastasis exhibited broad value ranges and substantial positive and negative effects in SHAP distributions, identifying them as pivotal predictors of PFS. Subsequent KM analysis of these 5 key predictors demonstrated that patients aged <63 years, those with signet ring cell carcinoma histology, elevated pretreatment proportion of CD19⁺ B cells, reduced pretreatment proportion of CD16⁺CD56⁺ NK cells, or presence of liver metastasis experienced significantly worse prognosis (Multimedia Appendix 8).

## Discussion

### Principal Results

In this study, we developed and compared 4 prognostic models using a comprehensive set of baseline parameters to predict PFS in patients with AGC receiving first-line immunochemotherapy. Our principal finding is that an ML model based on the RSF algorithm exhibited superior predictive performance over the LASSO-Cox, XGBoost, and survival-SVM models. Specifically, the RSF model exhibited the highest discriminatory power (as measured by C-index and AUC) and the greatest clinical utility (as determined by DCA) across both internal and temporal validation cohorts. Furthermore, the RSF-derived risk stratification system effectively stratified patients into distinct high- and low-risk prognostic groups, underscoring its potential utility in guiding individualized therapeutic decisions. Interpretability analysis subsequently identified 5 key predictors driving the model’s performance: age, histological subtype, pretreatment proportion of CD19⁺ B cells, pretreatment proportion of CD16⁺CD56⁺ NK cells, and the presence of liver metastasis. Collectively, these findings establish a robust and clinically applicable tool for personalized prognostic assessment in this patient population.

In-depth analysis of these predictors revealed that tumor-infiltrating lymphocytes and liver metastasis status significantly influenced prognosis. Our study observed that patients with an elevated pretreatment proportion of CD19⁺ B cells exhibited poorer outcomes. Analysis of the cohort demonstrated a mPFS of 7.4 (95% CI 6.67-8.9) months in the high CD19⁺ B cells subgroup compared with 15.9 (95% CI 10.9-upper limit not reached) months in the low CD19⁺ B cells subgroup. The function of B cells in cancer is highly heterogeneous, with distinct subsets capable of exerting either protumorigenic or antitumorigenic effects [33,34]. While the presence of B cells organized in tertiary lymphoid structure is often correlated with improved clinical outcomes and response to immunotherapy in many cancers [35], a substantial body of evidence also points to a protumorigenic B cell function. This negative role is frequently mediated by regulatory B cells (Bregs), which suppress antitumor immunity through the secretion of inhibitory cytokines like IL-10, IL-35, and TGF-β [36,37]. Our observation is consistent with studies specifically in GC, which have identified intratumoral Bregs (eg, CD19^+^CD24^hi^CD38^hi^ or CD19^+^CD24^hi^CD27^+^) that produce IL-10 [38]. Furthermore, IL-35-secreting B cells have been found to be elevated in patients with AGC and correlate with an accumulation of other immunosuppressive populations, such as regulatory T cells [39]. Therefore, it is plausible that the high CD19^+^ B cell population identified in our poor-prognosis cohort represents a dominance of these immunosuppressive Breg subsets, which dampen T cell effector function and contribute to therapeutic resistance. Conversely, other B cell subsets in the tumour microenvironment (TME) function as potent antigen-presenting cells to prime T cell responses or differentiate into plasma cells that produce tumor-specific antibodies [40]. The continued development and application of advanced analytical approaches, such as single-cell and spatial omics, are essential for comprehensively dissecting B cell heterogeneity and elucidating the precise mechanisms by which B cells modulate the immune response in GC.

NK cells are cytotoxic innate-like lymphocytes essential for tumor immunosurveillance, hardwired to recognize and kill stressed or malignant cells [41]. Evidence from mouse models confirms this, as long-term NK cell depletion results in increased tumor incidence and severity, demonstrating their fundamental role in suppressing tumor development [42]. NK cells deploy multiple antitumor mechanisms. Their activity is often perforin-dependent, highlighting the importance of direct cytotoxicity. Furthermore, NK cells express activating receptors like the high-affinity Fc receptor CD16, enabling them to mediate antibody-dependent cell-mediated cytotoxicity [43]. This function, critical for many antibody-based therapies, triggers the killing of antibody-coated cells and cytokine release. Beyond direct killing, recent work reveals a crucial “sentinel” function: NK cells detect nascent tumors and recruit dendritic cells to the TME, thereby mobilizing the adaptive immune response [44,45]. Clinically, numerous studies from The Cancer Genome Atlas correlate high NK cell infiltration with improved OS outcomes in diverse cancers, including melanoma, breast cancer, and GC [46-48].

Additionally, liver metastasis emerged as an independent adverse prognostic factor for the efficacy of first-line immunochemotherapy in AGC. KM curve analysis confirmed that patients with liver metastasis responded poorly to immunochemotherapy compared with those with distant metastases in other organs. Mechanistically, this is attributed to the formation of a highly immunosuppressive TME within the liver [49]. Numerous studies support this, revealing an increased presence of cancer-associated fibroblasts, myeloid-derived suppressor cell-like macrophages, tumor-associated macrophage-like macrophages, and naive T cells in liver metastasis. Conversely, conventional dendritic cells and effector CD8^+^ T cells were diminished [50,51]. This aligns with existing literature. Liver metastasis is known to upregulate immune checkpoint molecules like PD-1 or PD-L1, which can induce systemic exhaustion of tumor-specific CD8^+^ T cells and attenuate immunotherapy efficacy [52].

In summary, this study is the first to develop and rigorously validate an ML-based prognostic model specifically for patients with AGC receiving first-line immunochemotherapy. A key advantage of this model is its foundation on readily available, baseline clinical variables, offering a cost-effective and easily implementable prognostic tool in contrast to more complex omics-based assays. We demonstrated that the RSF model provides superior predictive performance and clinical utility, effectively stratifying patients into distinct high- and low-risk groups. This model is driven by key predictors identified in our analysis, including baseline immune cell proportions (CD19^+^ B cells and CD16^+^CD56^+^ NK cells) and the presence of liver metastasis. Consequently, the RSF model represents a robust tool that offers quantifiable decision support, holding significant potential for guiding individualized therapeutic strategies and enhancing clinical decision-making in this setting.

### Limitations and Future Work

Despite successful validation of the RSF model’s performance in predicting outcomes for patients with AGC receiving first-line immunochemotherapy via internal and temporal validation cohorts, several limitations warrant acknowledgment. First, the single-center, retrospective design is a primary constraint that may introduce selection bias. Our cohorts were derived from a single institution with specific patient demographics, referral patterns, and clinical management protocols. Consequently, the model may be overfit to these local characteristics, and its external validity remains to be determined. Second, the high prevalence of missing values for established biomarkers, notably PD-L1, MSI status, and TMB, presented a significant limitation. This data sparsity precluded a direct comparative analysis of the prognostic accuracy of our RSF model against these established biologically based predictors. Furthermore, it inhibited our ability to investigate whether the integration of these potent biomarkers could have further augmented the model’s predictive performance. Third, our findings are based on a multiple imputation approach, which assumes that data are missing at random, a potential source of bias if violated. However, the robustness of our main conclusions was confirmed by a sensitivity analysis, mitigating this concern.

Future research will focus on a multistage approach designed to rigorously validate, enhance, and ultimately translate our RSF model into clinical practice. The most immediate priority is to address the current limitation of generalizability. This requires rigorously testing the model’s performance and robustness in independent, prospective, and multicenter cohorts to definitively establish its external validity. Following successful validation, the next phase will involve enhancing the model’s predictive power by incorporating broader multiomics data, such as genomic, transcriptomic, or proteomic profiles. This integration not only promises to refine prognostic accuracy but may also uncover deeper biological insights into the mechanisms of immunochemotherapy response. Contingent upon this robust validation and enhancement, the final objective is the translation of the optimized model into an accessible clinical decision support tool. We envision the development of a user-friendly platform, such as a web-based calculator or a simplified score system. This would provide clinicians with a practical means to generate real-time, individualized predictions for prognosis and recurrence risk, thereby advancing personalized treatment strategies for patients with AGC.

### Conclusions

In conclusion**,** this study draws the following findings. First, 4 prognostic models were developed in this study, with comparative analyses demonstrating that the RSF model outperformed others in discriminative ability, calibration, and clinical utility. Second, SHAP analysis of the RSF model identified age, liver metastasis, histological subtype, and tumor-infiltrating lymphocytes as critical prognostic predictors for patients with AGC undergoing first-line immunochemotherapy. Third, this study is the first to use ML to construct a prognostic model for assessing treatment efficacy in patients with AGC receiving first-line immunochemotherapy. The results underscore the model’s significant clinical value in prognostic evaluation and therapeutic decision-making. This study provides methodological innovation and evidence-based medical rationale for advancing precision therapy in AGC, offering substantial potential for clinical translation.

## Appendix

Multimedia Appendix 1 Diagnostic plots of each variable after multiple imputation.

Multimedia Appendix 2 Sensitivity analysis evaluating the predictive performance in the complete-case cohort.

Multimedia Appendix 3 Demographics and clinicopathologic characteristics of the training, internal validation and temporal validation cohorts.

Multimedia Appendix 4 Results of univariable Cox proportional hazards regression analysis for progression-free survival in the training set.

Multimedia Appendix 5 Hyperparameter tuning.

Multimedia Appendix 6 RSF model and ranking of variable importance.

Multimedia Appendix 7 Continuous AUC curves for the four models: A represents the internal validation cohort, while B represents the temporal validation cohort.

Multimedia Appendix 8 Kaplan–Meier survival analysis based on age, pathological histological subtype, pre-treatment proportion of CD19⁺ B cells, pre-treatment proportion of CD16⁺CD56⁺ NK cells, and presence of liver metastasis.

## Abbreviations

AFP: alpha-fetoprotein
AGC: advanced gastric cancer
AUC: area under the curve
Breg: regulatory B cell
C-index: concordance index
DCA: decision curve analysis
DT: decision tree
GC: gastric cancer
HER2: human epidermal growth factor receptor 2
ICI: immune checkpoint inhibitor
KM: Kaplan-Meier
KS: Kolmogorov-Smirnov
LASSO: the least absolute shrinkage and selection operator
ML: machine learning
mPFS: median progression-free survival
MSI: microsatellite instability
mut/Mb: mutations per megabase
NK: natural killer
OS: overall survival
PD-1: programmed cell death protein 1
PD-L1: programmed death-ligand 1
PFS: progression-free survival
RECIST: Response Evaluation Criteria in Solid Tumors
ROC: receiver operating characteristic
RSF: random survival forest
SHAP: Shapley additive explanations
SVM: support vector machine
TMB: tumor mutation burden
TME: tumour microenvironment
XGBoost: extreme gradient boosting
