# Supplementary figures and images for: A Machine Learning Model Based on Clinical Factors to Predict the Efficacy of First-Line Immunochemotherapy for Patients With Advanced Gastric Cancer: Retrospective Study
Source: JMIR Med Inform. 2025 Dec 22;13:e82533. doi: 10.2196/82533 (PMC12770927; doi:10.2196/82533)

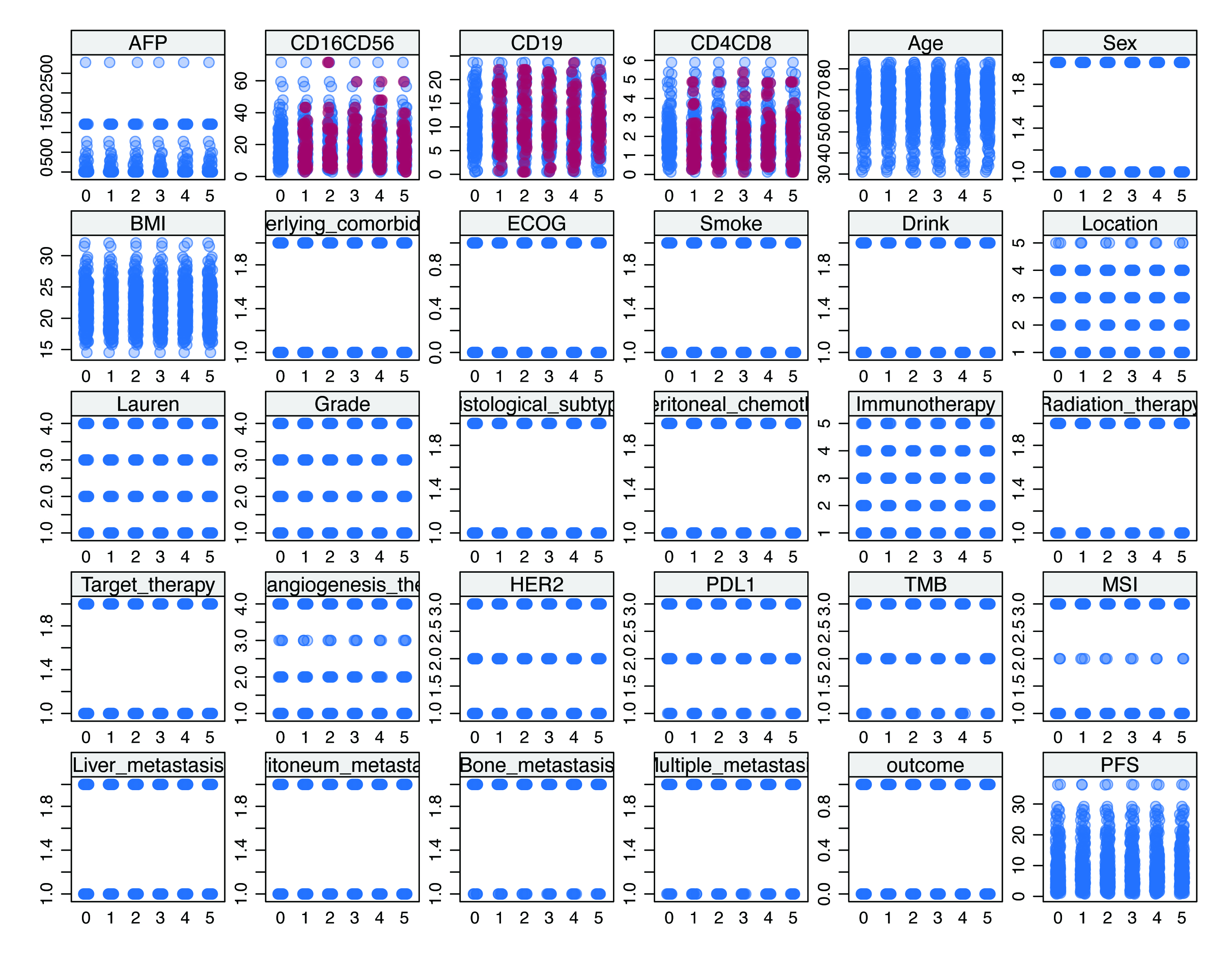

Supplement: Multimedia Appendix 1 [file medinform_v13i1e82533_app1.png]

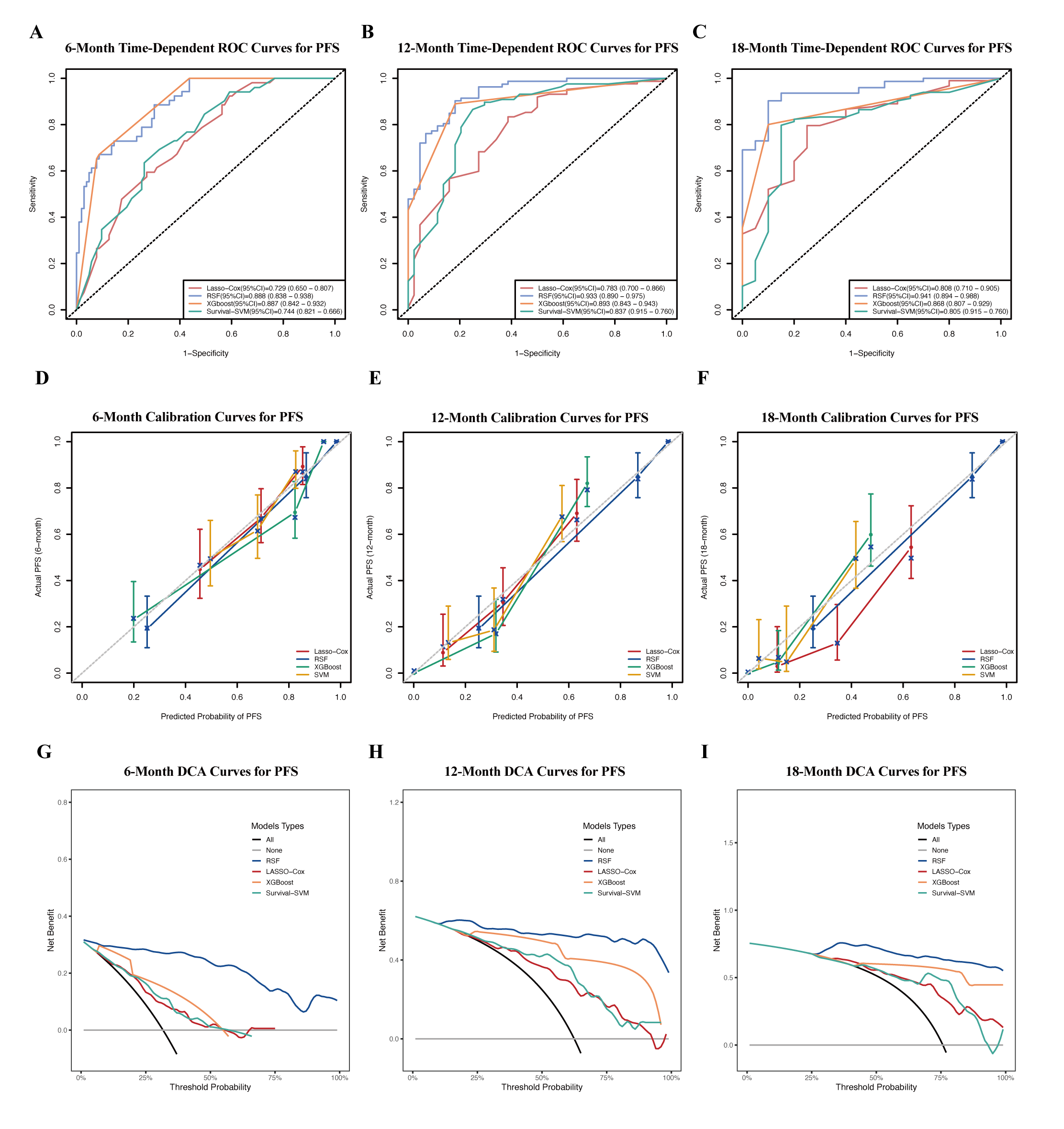

Supplement: Multimedia Appendix 2 [file medinform_v13i1e82533_app2.png]

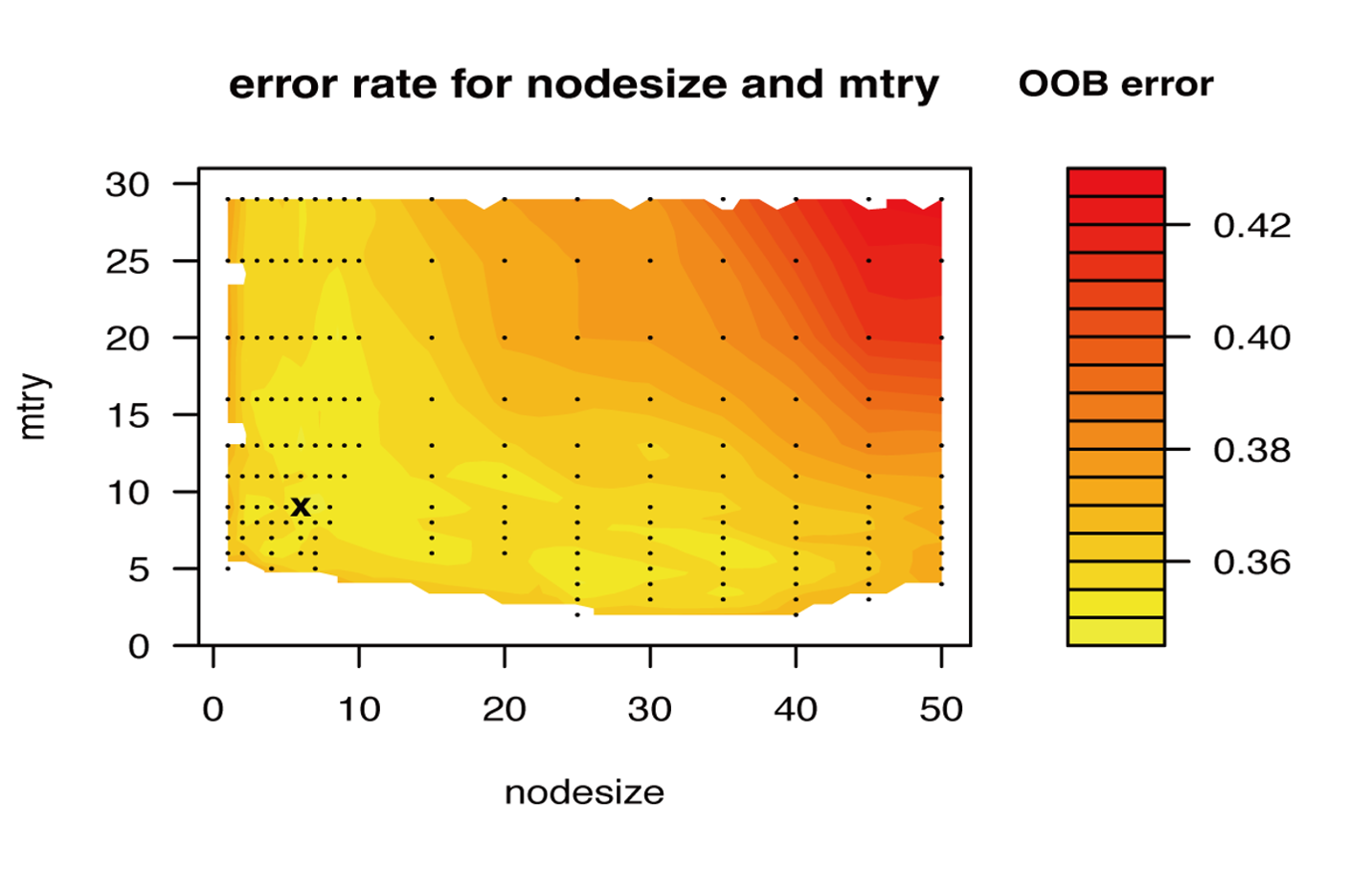

Supplement: Multimedia Appendix 5 [file medinform_v13i1e82533_app5.png]

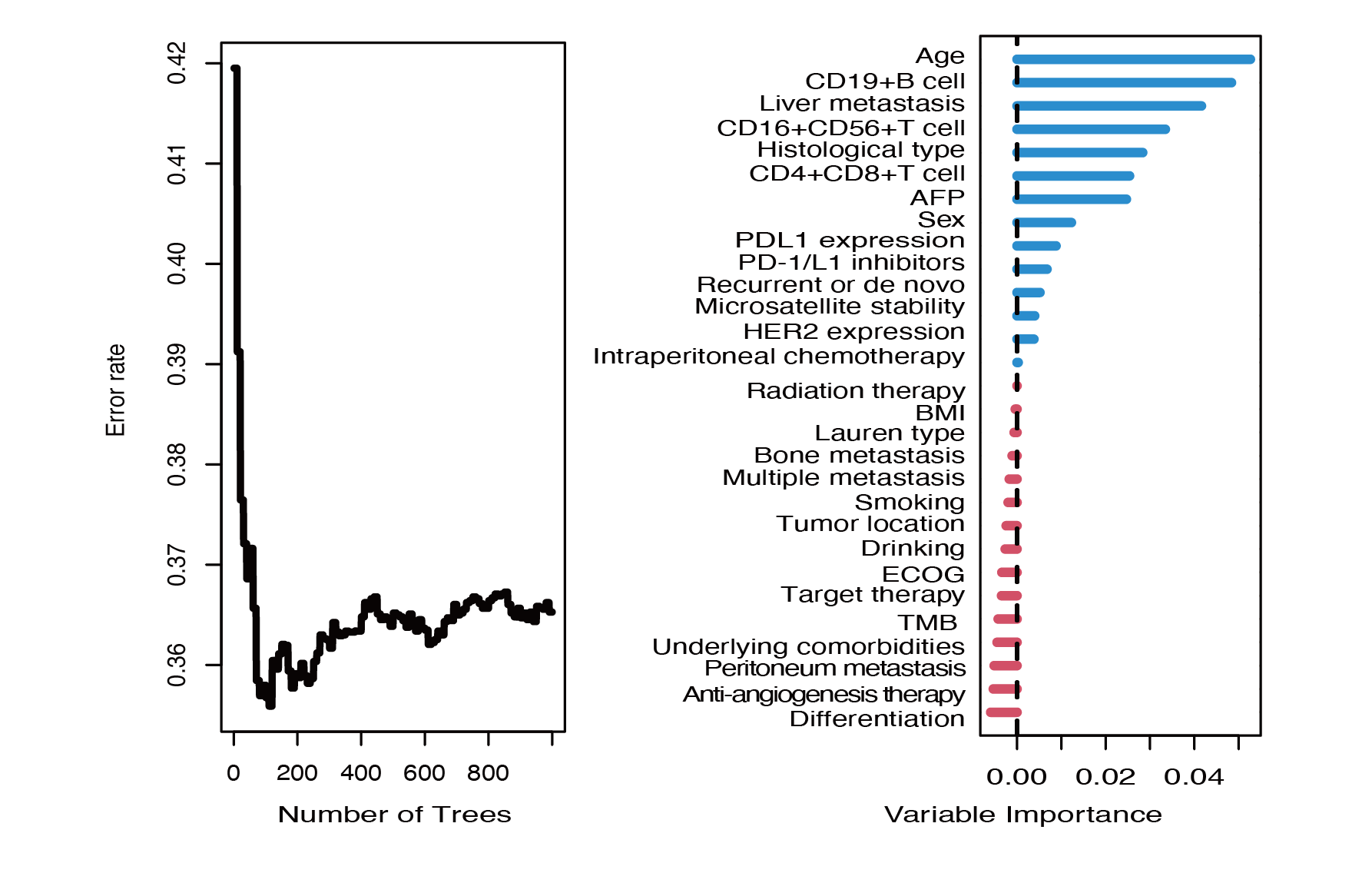

Supplement: Multimedia Appendix 6 [file medinform_v13i1e82533_app6.png]

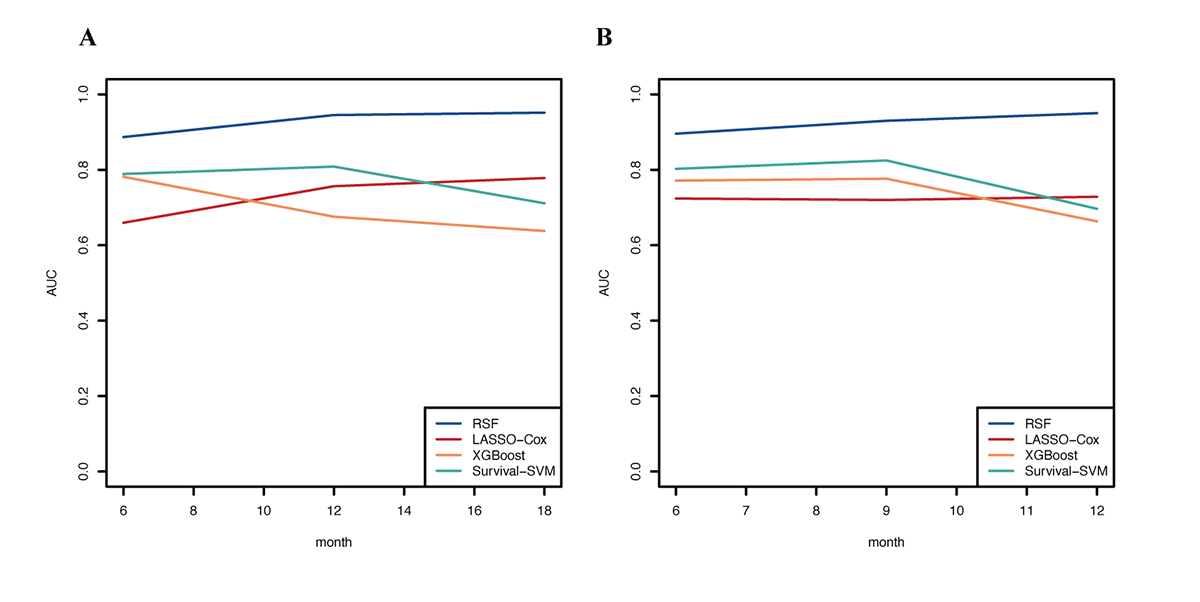

Supplement: Multimedia Appendix 7 [file medinform_v13i1e82533_app7.png]

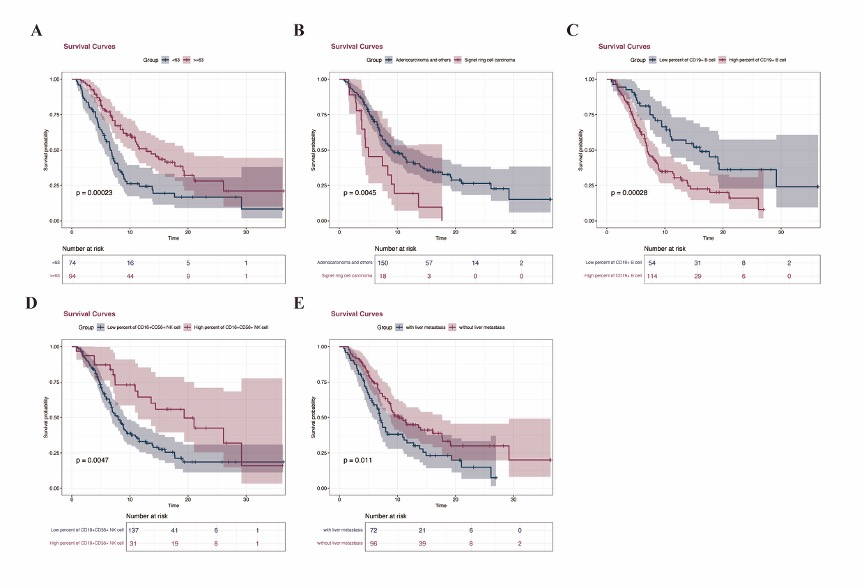

Supplement: Multimedia Appendix 8 [file medinform_v13i1e82533_app8.png]
