# Supplementary material for: A Machine Learning Model Based on Clinical Factors to Predict the Efficacy of First-Line Immunochemotherapy for Patients With Advanced Gastric Cancer: Retrospective Study
Source: JMIR Med Inform. 2025 Dec 22;13:e82533. doi: 10.2196/82533 (PMC12770927; doi:10.2196/82533)
Supplement: Multimedia Appendix 3 [file medinform_v13i1e82533_app3.docx]

**Multimedia Appendix 3**

Table S1 Demographics and clinicopathologic characteristics of the training, internal validation and temporal validation cohorts

| Characteristics | | Training Cohort  (N=168) | Internal Validation Cohort  (N=72) | Temporal Validation Cohort  (N=76) | *P*  value |
| --- | --- | --- | --- | --- | --- |
| **Age(Years)** | |  |  |  |  |
|  | <63 | 74(44.0%) | 31(43.1%) | 28(36.8%) | .563 |
|  | ≥63 | 94(56.0%) | 41(56.9%) | 48(63.2%) |  |
| **Sex** | |  |  |  |  |
|  | Male | 123(73.2%) | 55(76.4%) | 54(71.1%) | .761 |
|  | Female | 45(26.8%) | 17(23.6%) | 22(28.9%) |  |
| **BMI** | |  |  |  |  |
|  | Normal (<25) | 135(80.4%) | 60(83.3%) | 64(84.2%) | .725 |
|  | Overweight(≥25) | 33(19.6%) | 12(16.7%) | 12(15.8%) |  |
| **Underlying comorbidities** | |  |  |  |  |
|  | Yes | 58(34.5%) | 20(27.8%) | 26(34.2%) | .573 |
|  | No | 110(65.5%) | 52(72.2%) | 50(65.8%) |  |
| **ECOG PS** | |  |  |  |  |
|  | 0 | 91(54.2%) | 39(54.2%) | 45(59.2%) | .743 |
|  | 1 | 77(45.8%) | 33(45.8%) | 31(40.8%) |  |
| **Smoking** | |  |  |  |  |
|  | Yes | 47(28.0%) | 25(34.7%) | 26(34.2%) | .461 |
|  | No | 121(72.0%) | 47(65.3%) | 50(65.8%) |  |
| **Drinking** | |  |  |  |  |
|  | Yes | 36(21.4%) | 18(25.0%) | 24(31.6%) | .234 |
|  | No | 132(78.6%) | 54(75.0%) | 52(68.4%) |  |
| **Tumor location** | |  |  |  |  |
|  | Cardia of stomach and esophagogastric junction | 53(31.5%) | 23(31.9%) | 22(28.9%) | .814 |
|  | Fundus of stomach | 17(10.1%) | 2(2.8%) | 8(10.5%) |  |
|  | Body of stomach | 56(33.3%) | 28(38.9%) | 25(32.9%) |  |
|  | Antrum of stomach | 40(23.8%) | 18(25.0%) | 20(26.3%) |  |
|  | Pylorus of stomach | 2(1.2%) | 1(1.4%) | 1(1.3%) |  |
| **Lauren type** | |  |  |  |  |
|  | Intestinal type | 22(13.1%) | 19(26.4%) | 13(17.1%) | .216 |
|  | Mixed type | 17(10.1%) | 4(5.6%) | 10(13.2%) |  |
|  | Diffusion-type | 15(8.9%) | 5(6.9%) | 7(9.2%) |  |
|  | Unknown | 114(67.9%) | 44(61.1%) | 46(60.5%) |  |
| **Differentiation** | |  |  |  |  |
|  | G3 | 63(37.5%) | 20(27.8%) | 39(51.3%) | .034 |
|  | G2 | 15(8.9%) | 13(18.1%) | 10(13.2%) |  |
|  | G2-G3 | 28(16.7%) | 15(20.8%) | 9(11.8%) |  |
|  | Unknow | 62(36.9%) | 24(33.3%) | 18(23.7%) |  |
| **Histological subtype** | |  |  |  |  |
|  | Gastric adenocarcinoma | 150(89.3%) | 63(87.5%) | 60(78.9%) | .088 |
|  | Signet ring cell carcinoma | 18(10.7%) | 9(12.5%) | 16(21.1%) |  |
| **Intraperitoneal chemotherapy** | |  |  |  |  |
|  | Yes | 21(12.5%) | 9(12.5%) | 9(11.8%) | .989 |
|  | No | 147(87.5%) | 63(87.5%) | 67(88.2% |  |
| **PD-1/L1 inhibitors** | |  |  |  |  |
|  | Sintilimab | 101(60.1%) | 46(63.9%) | 68(89.5%) | .002 |
|  | Nivolumab | 25(14.9%) | 8(11.1%) | 3(3.9% |  |
|  | Tislelizumab | 20(11.9%) | 11(15.3%) | 4(5.3%) |  |
|  | Camrelizumab | 11(6.5%) | 5(6.9%) | 0(0) |  |
|  | other | 11(6.5%) | 2(2.8%) | 1(1.3%) |  |
| **Radiation therapy** | |  |  |  |  |
|  | Yes | 12(7.1%) | 3(4.2%) | 3(3.9%) | .496 |
|  | No | 156(92.9%) | 69(95.8%) | 73(96.1%) |  |
| **Target therapy** | |  |  |  |  |
|  | Yes | 49(29.2%) | 24(33.3%) | 12(15.8%) | .035 |
|  | No | 119(70.8%) | 48(66.7%) | 64(84.2%) |  |
| **Anti-angiogenesis therapy** | |  |  |  |  |
|  | Anlotinib | 21(12.5%) | 13(18.1%) | 4(5.3%) | .046 |
|  | Apatinib | 12(7.1%) | 6(8.3%) | 1(1.3%) |  |
|  | Other | 4(2.4%) | 0(0.0%) | 3(3.9%) |  |
|  | None | 131(78.0%) | 53(73.6%) | 68(89.5%) |  |
| **HER2 expression** | |  |  |  |  |
|  | Positive | 43(25.6%) | 19(26.4%) | 29(38.2%) | .003 |
|  | Negative | 26(15.5%) | 7(9.7%) | 20(26.3%) |  |
|  | Unknow | 99(58.9%) | 46(63.9%) | 27(35.5%) |  |
| **PD1/L1 expression** | |  |  |  |  |
|  | Positive | 13(7.7%) | 1(1.4%) | 13(17.1%) | .007 |
|  | Negative | 21(12.5%) | 5(6.9%) | 7(9.2%) |  |
|  | Unknow | 133(79.8%) | 66(91.7%) | 56(73.7%) |  |
| **TMB expression** | |  |  |  |  |
|  | High expression | 8(4.8%) | 2(2.8%) | 0(0) | .109 |
|  | Low expression | 18(10.7%) | 3(4.2%) | 10(13.2%) |  |
|  | Unknow | 142(84.5%) | 67(93.1%) | 66(86.8%) |  |
| **Microsatellite stability** | |  |  |  |  |
|  | MSS | 36(21.4%) | 9(12.5%) | 26(34.2%) | .027 |
|  | MSI-H | 2(1.2%) | 1(1.4%) | 0(0) |  |
|  | Unknow | 130(77.4%) | 62(93.1%) | 50(65.8%) |  |
| **Liver metastasis** | |  |  |  |  |
|  | Yes | 72(42.9%) | 37(51.4%) | 33(43.4%) | .455 |
|  | No | 96(57.1%) | 35(48.6%) | 43(56.6%) |  |
| **Peritoneum metastasis** | |  |  |  |  |
|  | Yes | 45(26.8%) | 15(20.8%) | 19(25%) | .621 |
|  | No | 123(73.2%) | 57(79.2%) | 57(75%) |  |
| **Bone metastasis** | |  |  |  |  |
|  | Yes | 8(4.8%) | 3(4.2%) | 6(7.9%) | .528 |
|  | No | 160(95.2%) | 69(95.8%) | 70(92.1%) |  |
| **Multiple metastasis** | |  |  |  |  |
|  | Yes | 11(6.5%) | 2(2.8%) | 6(7.9%) | .388 |
|  | No | 157(93.5%) | 70(97.2%) | 70(92.1%) |  |
| **AFP(ng/μL)** | |  |  |  |  |
|  | <1.9 | 35(20.8%) | 17(23.6%) | 14(18.4%) | .740 |
|  | ≥1.9 | 133(79.2%) | 55(76.4%) | 62(81.6%) |  |
| **The proportion of CD16^+^CD56^+^ NK cell** | |  |  |  |  |
|  | <27.41 | 137(81.5%) | 54(75.0%) | 55(72.4%) | .224 |
|  | ≥27.41 | 31(18.5%) | 18(25.0%) | 21(27.6%) |  |
| **The proportion of CD19^+^B cell** | |  |  |  |  |
|  | <12.04 | 54(32.1%) | 19(26.4%) | 32(42.1%) | .116 |
|  | ≥12.04 | 114(67.9%) | 53(73.6%) | 44(57.9%) |  |
| **The proportion of CD4^+^/CD8^+^T cell** | |  |  |  |  |
|  | <2.66 | 110(65.5%) | 54(66.7%) | 51(67.1%) | .964 |
|  | ≥2.66 | 58(34.5%) | 24(33.3%) | 25(32.9%) |  |
| **PFS(months)** | |  |  |  |  |
|  | Median(IQR) | 7.27[4.71,12.70] | 7.30[4.58,12.08] | 6.05[4.83,7.68] | .031 |
