# Supplementary material for: A Machine Learning Model Based on Clinical Factors to Predict the Efficacy of First-Line Immunochemotherapy for Patients With Advanced Gastric Cancer: Retrospective Study
Source: JMIR Med Inform. 2025 Dec 22;13:e82533. doi: 10.2196/82533 (PMC12770927; doi:10.2196/82533)
Supplement: Multimedia Appendix 4 [file medinform_v13i1e82533_app4.docx]

**Multimedia Appendix 4**

Table S2 Results of univariable Cox proportional hazards regression analysis for progression-free survival in the training set.

| Characteristics | | Univariable  Cox regression | |  |
| --- | --- | --- | --- | --- |
|  |  | Hazard Ratio(95%) | *P* value |  |
| **Age(years)** | |  |  |  |
|  | ≥63VS<63 | 0.49(0.34-0.72) | <.001 |  |
| **Sex** | |  |  |  |
|  | Female VS Male | 1.36(0.89-2.09) | .159 |  |
| **BMI** | |  |  |  |
|  | ≥25 VS <25 | 0.89(0.55-1.46) | .654 |  |
| **Underlying comorbidities** | |  |  |  |
|  | Without VS with | 0.95(0.63-1.43) | .802 |  |
| **ECOG PS** | |  |  |  |
|  | 1 VS 0 | 0.72(0.49-1.06) | .095 |  |
| **Smoking** | |  |  |  |
|  | No VS Yes | 1.31(0.84-2.04) | .237 |  |
| **Drinking** | |  |  |  |
|  | No VS Yes | 1.21(0.74-1.99) | .449 |  |
| **Tumor location** | |  |  |  |
|  | Fundus of stomach VS Cardia of stomach and esophagogastric junction | 0.95(0.47-1.92) | .881 |  |
|  | Body of stomach VS Cardia of stomach and esophagogastric junction | 1.17(0.73-1.88) | .504 |  |
|  | Antrum of stomach VS Cardia of stomach and esophagogastric junction | 1.05(0.62-1.78) | .852 |  |
|  | Pylorus of stomach VS Cardia of stomach and esophagogastric junction | 1.60(0.38-6.68) | .520 |  |
| **Lauren** | |  |  |  |
|  | Mixed type VS Intestinal type | 1.40(0.65-3.03) | .393 |  |
|  | Diffusion-type VS Intestinal type | 1.83(0.82-4.09) | .143 |  |
|  | Unknown VS Intestinal type | 1.12(0.62-2.02) | .716 |  |
| **Differentiation** | |  |  |  |
|  | G2 VS G3 | 1.07(0.52-2.24) | .848 |  |
|  | G2-G3 VS G3 | 1.15(0.65-2.03) | .636 |  |
|  | Unknown VS G3 | 1.07(0.68-1.67) | .767 |  |
| **Histological type** | |  |  |  |
|  | Signet ring cell carcinoma VS Gastric adenocarcinoma | 2.18(1.26-3.78) | .006 |  |
| **Intraperitoneal chemotherapy** | |  |  |  |
|  | No VS Yes | 0.71(0.41-1.23) | .222 |  |
| **PD-1 /L1 inhibitors** | |  |  |  |
|  | Nivolumab VS Sintilimab | 0.80(0.45-1.41) | .438 |  |
|  | Tislelizumab VS Sintilimab | 1.70(0.99-2.93) | .055 |  |
|  | Camrelizumab VS Sintilimab | 0.63(0.28-1.41) | .258 |  |
|  | Other VS Sintilimab | 0.89(0.40-1.95) | .767 |  |
| **Radiotherapy** | |  |  |  |
|  | No VS Yes | 0.87(0.44-1.73) | .692 |  |
| **Antiangiogenesis therapy** | |  |  |  |
|  | Apatinib VS Anlotinib | 1.16(0.53-2.54) | .710 |  |
|  | Other VS Anlotinib | 0.37(0.05-2.83) | .340 |  |
|  | Unknown VS Anlotinib | 0.81(0.47-1.41) | .464 |  |
| **Target Therapy** | |  |  |  |
|  | No VS Yes | 0.94(0.63-1.42) | .772 |  |
| **AFP(ng/μL)** | |  |  |  |
|  | ≥1.9VS<1.9 | 2.17(1.27-3.72) | .005 |  |
| **The proportion of CD16^+^CD56^+^T cell** | |  |  |  |
|  | ≥27.41 VS <27.41 | 0.47(0.27-0.80) | .006 |  |
| **The proportion of CD19^+^B cell** | |  |  |  |
|  | ≥12.04 VS <12.04 | 2.23(1.43-3.48) | <.001 |  |
| **The proportion of CD4^+^/CD8^+^T cell** | |  |  |  |
|  | ≥2.66 VS <2.66 | 0.60(0.39-0.91) | .017 |  |
| **HER2 expression** | |  |  |  |
|  | Negative VS Positive | 0.83(0.45-1.56) | .573 |  |
|  | Unknow VS Positive | 0.75(0.48-1.17) | .205 |  |
| **PD-L1 expression** | |  |  |  |
|  | Negative VS Positive | 1.25(0.50-3.13) | .639 |  |
|  | Unknow VS Positive | 1.26(0.58-2.74) | .557 |  |
| **TMB expression** | |  |  |  |
|  | Low expression VS High expression | 3.35(1.06-10.63) | .040 |  |
|  | Unknow VS High expression | 2.12(0.75-6.01) | .156 |  |
| **Microsatellite stability** | |  |  |  |
|  | MSI-H VS MSS | 1.63(0.22-12.17) | .632 |  |
|  | Unknow VS MSS | 0.86(0.55-1.36) | .524 |  |
| **Liver metastasis** | |  |  |  |
|  | Without VS With | 0.61(0.42-0.90) | .012 |  |
| **Peritoneum metastasis** | |  |  |  |
|  | Without VS With | 1.13(0.71-1.78) | .610 |  |
| **Bone metastasis** | |  |  |  |
|  | Without VS With | 0.76(0.33-1.74) | .518 |  |
| **Multiple metastasis** | |  |  |  |
|  | Without VS With | 0.76(0.37-1.57) | .457 |  |
